# S11 Improving knowledge co-creation and participation in physical activity promotion: The cooperative planning approach

**DOI:** 10.1093/eurpub/ckac093.054

**Published:** 2022-08-29

**Authors:** Peter Gelius, Raluca Sommer, Susanne Ferschl, Maike Till, Karim Abu-Omar, Jana Semrau, Nathalie Helsper, Simone Kohler, Lea Dippon, Klaus Pfeifer, Alfred Rütten, Johanna Popp, Johannes Carl, Eva Grüne, Christina Müller, Holger Hassel, Dorothee Altmeier, Annika Frahsa, Ansgar Thiel

**Affiliations:** 1 Department of Sport Science and Sport, FAU Erlangen-Nürnberg, Erlangen, Germany; 2 Institut für angewandte Gesundheitswissenschaften, Coburg University of Applied Sciences and Arts, Coburg, Germany; 3 Institut für Sportwissenschaft, Universität Tübingen, Tübingen, Germany

**Keywords:** health promotion theory, participatory approaches, knowledge co-creation, scaling-up, sustainability

## Abstract

A central problem of current efforts to promote health and physical activity (PA) is that many successful projects remain stuck in the demonstration phase and are not implemented successfully at scale. The use of participatory and/or co-creation approaches has been suggested to avoid this ?pilot project trap? and better adapt interventions to target group needs and setting specificities.

This symposium intends to introduce to an international audience a particular participatory concept that has become increasingly popular in PA promotion in Germany in recent years: The Cooperative Planning approach has been successfully used in sport facility planning, local and regional PA policy development, and various settings of PA promotion (incl. kindergartens, schools, vocational training, and communities). The workshop will shed light on the theoretical background and methodology of Cooperative Planning as well as its specific application in select settings.

The first presentation will introduce the concept of Cooperative Planning, outline potential areas of application, and compare it with other popular participatory and co-creation approaches in PA promotion. Following this, we will provide evidence from ongoing projects employing the approach to promote PA in kindergartens (Presentation 2) and in the community setting (Presentation 3). Presentation 4 will introduce an example from the retirement home setting and also highlight ways of combining Cooperative Planning with other approaches such as photovoice and participatory evaluation. The final presentation will provide an outlook on the future extension of the concept by introducing the idea of the Practice Dive, which may be used to further optimize knowledge co-creation between researchers and practitioners.

A closer look at the Cooperative Planning approach is both timely and relevant for an international audience for a number of reasons: Conceptually, Cooperative Planning is a theory-based framework that combines ideas of participation and co-creation for PA into an innovative whole-of-system approach. From a practical PA promotion perspective, it transcends many existing techniques by focusing both on engaging multipliers and members of the target group, and by involving all of them in the decisive intervention development process (e.g. rather than only via opinion polls or focus groups). This symposium will allow us to combine evidence from four different projects, highlighting both the specificities of working in different settings as well as different aspects and possible extensions of the Cooperative Planning approach.

